# Mitral Valve Transcatheter Edge-to-Edge Repair (MV-TEER) in Patients with Secondary Mitral Regurgitation Improves Hemodynamics, Enhances Renal Function, and Optimizes Quality of Life in Patients with Advanced Renal Insufficiency

**DOI:** 10.3390/biomedicines12112648

**Published:** 2024-11-20

**Authors:** Birgit Markus, Julian Kreutz, Giorgios Chatzis, Styliani Syntila, Jannis Kuchenbuch, Charlotte Mueller, Maryana Choukeir, Bernhard Schieffer, Nikolaos Patsalis

**Affiliations:** Department of Cardiology, Angiology, and Intensive Care Medicine, University Hospital, Philipps University of Marburg, Baldinger Str., 35043 Marburg, Germany; birgit.markus@staff.uni-marburg.de (B.M.); kreutzj@staff.uni-marburg.de (J.K.); chatzis@staff.uni-marburg.de (G.C.); syntila@med.uni-marburg.de (S.S.); kuchenbu@students.uni-marburg.de (J.K.); charlotte.mueller96@gmail.com (C.M.); mchoukei@med.uni-marburg.de (M.C.); bernhard.schieffer@staff.uni-marburg.de (B.S.)

**Keywords:** MV-TEER, expanded hemodynamics, renal perfusion, renal function, quality of life

## Abstract

Background/Objectives: Secondary mitral regurgitation (MR) is a common valvular heart disease burdening the prognosis of patients with co-existing chronic heart failure. Transcatheter edge-to-edge mitral valve repair (MV-TEER) is a minimally invasive treatment option for high-risk patients. However, the effects of MV-TEER on expanded hemodynamics, tissue perfusion, and quality of life, particularly in patients with advanced renal failure, remain underexplored. Methods: This prospective, single-center study evaluated the impact of MV-TEER on hemodynamics, renal function, and quality of life in 45 patients with severe MR. Non-invasive bioimpedance monitoring with NICaS^®^ was used to assess hemodynamics pre- and 3–5 days post-procedure. Quality of life was assessed using the EQ-5D-3L questionnaire before and 3 months post-procedure. For further analysis, patients were divided into subgroups based on the estimated baseline glomerular filtration rate (eGFR < 35 mL/min vs. eGFR ≥ 35 mL/min). Results: A significant reduction in systemic vascular resistance (SVR; *p* = 0.003) and an increase in eGFR (*p* = 0.03) were observed in the entire cohort after MV-TEER, indicating improved tissue perfusion. Notably, particularly patients with eGFR < 35 mL/min showed a significant increase in cardiac output (CO; *p* = 0.035), cardiac index (CI; *p* = 0.031), and eGFR (*p* = 0.018), as well as a reduction in SVR (*p* = 0.007). Consistent with these findings, quality of life significantly improved, with the EQ-5D-3L index and EQ-VAS score increasing from 0.44 to 0.66 (*p* < 0.001) and from 51.7% to 62.9% (*p* < 0.001).

## 1. Introduction

Mitral regurgitation (MR) is the third most common valvular heart disease affecting over 24 million people worldwide. The most prevalent cause of MR is secondary or functional, primarily driven by left ventricular dilation as an underlying pathomechanism, accounting for approximately 65% of clinically relevant MR cases [1]. The second most common cause of MR is mitral valve prolapse, which underlies primary MR [2]. A currently high and further increasing number of patients with coronary artery disease (CAD) or congestive heart failure are at significant risk for developing secondary MR, further worsening their prognosis [3,4,5]. Moreover, the occurrence of severe MR in patients with chronic heart failure (CHF) is accompanied by significantly reduced survival [6].

The pathophysiology of secondary MR is complex. In the case of severe MR, volume overload of the left ventricle leads to left ventricular dilation, and the regurgitated volume causes pulmonary vein congestion with consecutive pulmonary edema leading to respiratory insufficiency. Furthermore, chronic pulmonary vein congestion burdens right ventricular function, increasing the mean pulmonary artery pressure which ultimately results in chronic pulmonary hypertension, further deteriorating right ventricular function [7,8]. As a consequence, venous congestion worsens tissue and organ perfusion, leading to impaired organ function, and ultimately resulting in liver or renal failure [9,10,11].

For severe, symptomatic secondary MR, current guidelines recommend first-line treatment through management of the underlying disease and the optimization of medical therapy. Interventional procedures, and in some cases, surgical repair of the mitral valve, are advised if patients remain symptomatic despite optimal medical treatment [12]. Multimorbidity in elderly patients is accompanied by a significantly higher risk for perioperative complications. For these patients, current guidelines recommend mitral valve transcatheter edge-to-edge repair (MV-TEER) to relieve symptoms and improve quality of life and outcomes [12].

However, these patients often present with complex conditions involving multiple interacting pathophysiological factors. Although numerous studies have investigated MV-TEER, highlighting its significant prognostic benefit, to our knowledge no studies have specifically examined its impact on expanded hemodynamics and end-organ perfusion. This limits the understanding of the changing pathophysiology in these complex and fragile patients before and after MV-TEER.

Therefore, this study aimed to evaluate the impact of MV-TEER on hemodynamics using non-invasive bioimpedance monitoring with NICaS^®^, an approved and easy-to-use bedside device that provides precise hemodynamic assessments [13,14,15,16,17]. Moreover, the study focused on evaluating correlations of MV-TEER with organ function and quality of life. By considering patients’ comorbidities, particularly the prognostic relevant renal function [13,14,15,16], this approach will help to optimize and tailor peri-procedural treatment strategies.

## 2. Materials and Methods

### 2.1. Study Design

This single-center prospective clinical study was performed over 1 year (June 2023–June 2024) at the University Hospital of Marburg, Germany. Data from 45 patients electively admitted for MV-TEER were included. All patients received optimal medical treatment prior to the procedure. Inclusion criteria were age ≥ 18 years, signed informed consent, and the presence of MV regurgitation grade 3 or 4 with a given indication for TEER according to the current guidelines [17]. During the in-hospital stay, documentation and measurements of various therapy-affiliated parameters were carried out at two different time points (T1 and T2). The first measurement (T1) was taken on the day of admission, approximately 24 h before the start of the TEER procedure. The second measurement (T2) was conducted 3–5 days after the TEER procedure, before discharge. The documented parameters included non-invasive hemodynamic bioimpedance measurements using the NICaS^®^ system (cardiac output; CO, cardiac index; CI and systemic vascular resistance; SVR), vital signs (heart rate, SAP, DAP, MAP), standard blood laboratory parameters (e.g., creatinine, estimated glomerular filtration rate (eGFR), NT proBNP), echocardiographic parameters, and relevant medical history data. For calculation of the eGFR, the MDRD (*Modification of Diet in Renal Disease*) formula was used, including creatinine, age, and sex.

### 2.2. NICaS^®^ Device and Procedure

The NICaS^®^ whole body electrical bio-impedance monitoring system (NIMedical, Israel Advanced Technology Industries, Hertzliya Pituach 4676672, Israel), which is FDA-approved and CE-certified, was utilized for non-invasive hemodynamic monitoring. Validation studies comparing NICaS^®^ to Swan-Ganz- and PICCO^®^-catheterization techniques have recently been published [18,19,20,21,22]. By combining pulse contour analysis with the Granov–Goor Index (GGI), derived from systolic time intervals (STI), NICaS^®^ enables easy-to-use bedside monitoring of cardiac function by displaying, e.g., the cardiac output (CO), cardiac index (CI), and systemic vascular resistance (SVR).

### 2.3. Impact of MV-TEER on Quality of Life (EQ-5D-3L Questionnaire)

To assess the impact of MV-TEER on quality of life during 3-month follow-up, the EQ-5D-3L questionnaire was incorporated into patient management (pre-procedure vs. 3 months post-procedure). The EQ-5D-3L questionnaire is comprised of 5 simple questions with 5 possible answers regarding mobility, self-care, usual activities, pain/discomfort, and anxiety/depression, as well as an additional visual analog scale (VAS) documenting the respondents’ self-estimate of health state with a value range from 0% representing the worst possible health state to 100% reflecting a perfect health status [23]. In addition to the VAS, an EQ-5D-3L index value was calculated based on responses to the five aforementioned questions, using index value sets validated specifically for Germany. This index ranges from 0 (worst health status) to 1 (best health condition) [24].

### 2.4. Study Objectives

The primary endpoint of the study was the impact of MV-TEER on hemodynamics and tissue perfusion in the overall cohort during short-term follow-up (pre-procedure vs. 3–5 days post-procedure).

Secondary endpoints were defined as the effects of MV-TEER on hemodynamics based on documented renal function (eGFR < 35 mL/min vs. eGFR ≥ 35 mL/min at T1 and T2). Additionally, the impact of MV-TEER on quality of life during three-month follow-up (3M-FU) was analyzed.

### 2.5. Statistical Analysis

Data were displayed as absolute variables and percentages (%) for categorical variables and either median with interquartile range (IQR, 25–75th percentile) or mean with SD according to variables’ distributions. Normality was determined by implementing Shapiro–Wilk, Pearson as well as Kolmogorov–Smirnov test. After evaluations of normal distribution, the Student *t*-test, the Mann–Whitney U test, or the Wilcoxon test were conducted to test for differences between the various characteristics. For categorical variables, the Fisher exact test or chi-square test was implemented, as appropriate. Linear regression analysis was performed to test for associations of hemodynamic values. All analyses were performed with SPSS version 28 (IBM, New York, NY, USA) and GraphPad Prism version 8.0 (GraphPad Software, San Diego, CA, USA). A two-sided *p*-value of less than 0.05 was defined as statistically significant.

## 3. Results

### 3.1. Overall Cohort

#### 3.1.1. Baseline Characteristics

The mean age of the overall cohort was 78.67 ± 6.38 years, 60% were male, and 40% were female. The average left ventricular ejection fraction was 55%. In total, 55.6% of the patients were accompanied by a third-grade mitral regurgitation, and in 44.4% of the patients, a fourth-grade mitral regurgitation was diagnosed. In total, 64.4% of the patients had a known chronic kidney disease (CKD). CKD was classified based on the evaluation of baseline (hospital admission) creatinine levels, as outlined in the current KDIGO guidelines [25]. To the best of our knowledge, impaired renal function in patients with CKD was due to cardio-renal syndrome. No evidence of an underlying primary kidney disease was noted in the patients’ histories. The demographics and baseline characteristics (on admission) of 45 patients undergoing MV-TEER are displayed in Table 1.

#### 3.1.2. Hemodynamics, Tissue Perfusion, and Renal Function

Following MV-TEER, no significant changes in SAP, DAP, or MAP were observed during the short-term follow-up period of 3–5 days post-procedure (T1 vs. T2). However, CO increased from the baseline 4.13 [3.09–5.38] L/min (T1) to 4.3 [3.86–5.69] L/min (T2) (*p* = 0.025) and CI increased from the baseline 2.31 [1.82–2.78] L/min/m^2^ to 2.44 [2.11–2.82] L/min/m^2^, *p* = 0.032. Furthermore, SVR dropped significantly from the baseline 1596 [1177–2132] N × s/m^5^ (T1) to 1427 [1148–1725] N × s/m^5^ (T2) (*p* = 0.003) indicating a decreased cardiac afterload resulting in improved tissue perfusion. Accordingly, a significant increase in eGFR from 46.26 ± 21.56 mL/min (T1) to 50.38 ± 21.34 (T2) (*p* = 0.03) was documented. Finally, in the overall cohort, the median MR grade was substantially reduced from grade 3 (T1) to grade 1 (T2), *p* < 0.001 (Table 2). Linear regression analysis revealed a positive association of MAP with SVR at T1 (*p* = 0.036, 95% regression coefficient B 19.17, CI 1.321–37.015) and at T2 (*p* = 0.024, 95% regression coefficient B 33.1, CI 4.48–61.7) (Tables S1 and S2).

### 3.2. Impact of MV-TEER in Relation to Renal Function

#### 3.2.1. Baseline Characteristics in eGFR Subgroups

**Figure 1:**
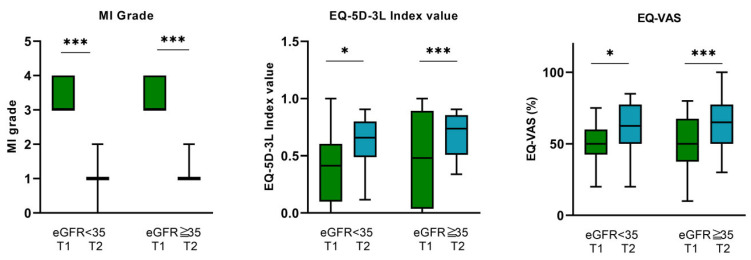
Mitral regurgitation grade before (T1) and after (T2) MV-TEER, EQ-5D-5L index value, and EQ-VAS before (T1) and 3 months after MV-TEER (3M-FU) in the eGFR < 35 mL/min and eGFR ≥ 35 mL/min subgroups. Abbreviations: MI: mitral insufficiency, eGFR: estimated glomerular filtration. * *p* < 0.05, *** *p* < 0.001.

At T1, 16 patients presented with an eGFR < 35 mL/min, while 29 patients had an eGFR ≥ 35 mL/min. Accordingly, patients with an eGFR < 35 mL/min exhibited significantly higher NT-proBNP levels at T1 compared to those with an eGFR ≥35 mL/min (8513 pg/mL [3859–15,356] vs. 2046 pg/mL [1378–4805], *p* < 0.001), suggesting more compromised cardiac function. Furthermore, the eGFR ≥ 35 mL/min subgroup was accompanied by a higher BMI (26.1 ± 4.4 vs. 26.8 ± 5.3, *p* = 0.041) and a higher hemoglobin value (111.9 ± 18.5 vs. 117.7 ± 18.1 g/L, *p* = 0.007), while patients of the eGFR < 35 mL/min subgroup had a higher rate of ICD implantations (4 (25%) vs. 1 (3.4%), *p* = 0.028). In both eGFR subgroups, MR could be significantly reduced (Table 1, Figure 1). No other relevant differences in baseline characteristics and comorbidities between these subgroups were documented (Table 1).

#### 3.2.2. Hemodynamics, Tissue Perfusion, and Renal Function

**Figure 2:**
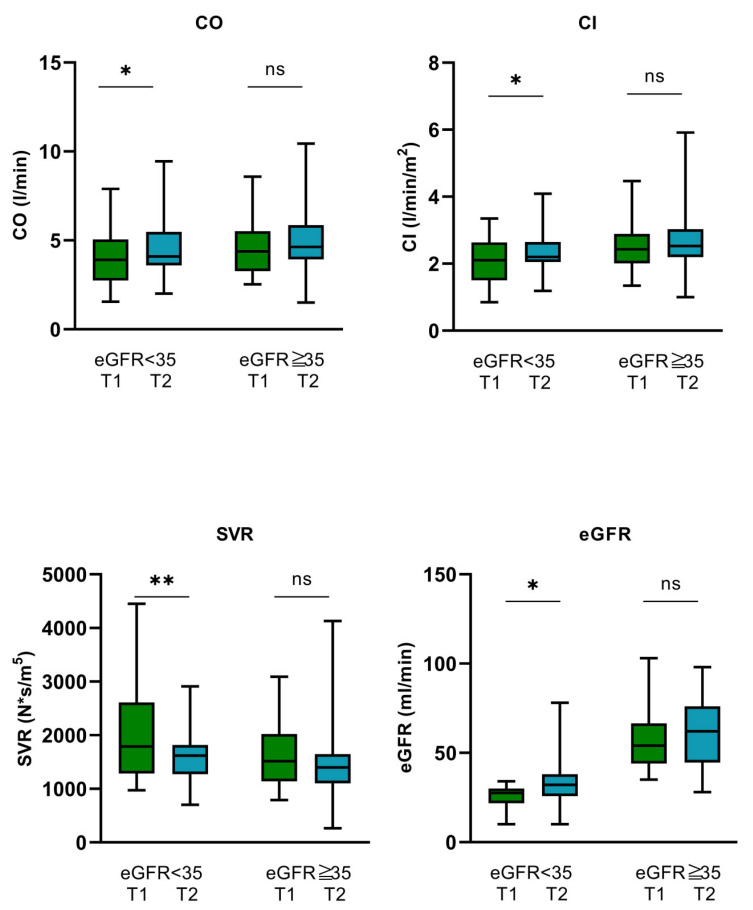
Hemodynamics and renal function before and after MV-TEER in the eGFR < 35 mL/min and eGFR > 35 mL/min subgroups. (CO: cardiac output, CI: cardiac index, SVR: systemic vascular resistance, eGFR: estimated glomerular filtration rate.) * *p* < 0.05, ** *p* < 0.01, ns: not significant.

In both eGFR subgroups, no significant alterations in SAP, DAP, MAP, and heart rate (HR) could be observed before and after MV-TEER (Table 3). However, patients with eGFR < 35 mL/min showed a significant improvement in CO and CI from 3.94 ± 1.6 (T1) to 4.47 ± 1.72 L/min (T2) (*p* = 0.035) and from 2.07 ± 0.69 (T1) to 2.34 ± 0.68 L/min/m^2^ (T2) (*p* = 0.031), respectively. Moreover, in this patient group, SVR dropped remarkably from 1791 N × s/m^5^ [1285–2612] (T1) to 1618 N × s/m^5^ [2205–5654] (T2) (*p* = 0.007). eGFR improved from baseline 25.63 ± 6.54 mL/min (T1) to 33.81 ± 16.05 mL/min (T2) (*p* = 0.018) indicating significantly improved hemodynamics and tissue and renal perfusion during short-term follow-up after MV-TEER. In contrast, no significant alterations of CO, CI, SVR, and eGFR could be observed for patients with eGFR ≥ 35 mL/min during a 3–5-day follow-up (Figure 2, Table 3).

### 3.3. Impact of MV-TEER on Quality of Life—EQ-5D-3L and EQ-VAS Questionnaire

#### 3.3.1. Overall Cohort

According to the results of the EQ-5D-3L questionnaire, a significant improvement in quality of life was observed after MV-TEER, with a comparison made between pre-procedure measurements and those taken at the 3-month follow-up. The EQ-5D-3L index value improved significantly from 0.44 ± 0.39 to 0.66 ± 0.20 (*p* < 0.001). Furthermore, EQ-VAS increased significantly from 51.7 ± 0.18% prior MV-TEER to 62.9 ± 0.17% (*p* < 0.001) 3 months after the procedure (Table 4).

#### 3.3.2. eGFR < 35 mL/min Subgroup

For patients with an eGFR < 35 mL/min at baseline, a significant improvement in quality of life after MV-TEER was also observed. The EQ-5D-3L index value increased from a baseline of 0.527 ± 0.13 to 0.61 ± 0.19 (*p* = 0.034), while the EQ-VAS increased from 51.6 ± 0.14% pre-procedure to 60.6 ± 0.18% at the 3-month follow-up (*p* = 0.015) (Figure 1, Table 4).

#### 3.3.3. eGFR ≥ 35 mL/min Subgroup

Patients within the eGFR ≥ 35 mL/min subgroup were also accompanied by a substantial improvement in quality of life after MV-TEER. In this subgroup, the EQ-5D-3L index value improved from 0.475 ± 0.4 before TEER to 0.68 ± 0.19 (*p* < 0.001) 3 months after MV-TEER. EQ-VAS also increased from 51.8 ± 0.17% to 64 ± 0.17%, *p* < 0.001 (Figure 1, Table 4).

## 4. Discussion

In this study, to our knowledge, we demonstrated for the first time that MV-TEER significantly improves expanded hemodynamics, including CO and CI, while reducing SVR. Increased organ perfusion after MV-TEER appears to improve renal function and overall quality of life. In particular, patients with reduced renal function (eGFR < 35 mL/min) may benefit more and show more significant improvements compared to those patients with preserved renal function (eGFR ≥ 35 mL/min).

MV-TEER is an established procedure for treating severe symptomatic MR initially intended for patients unable to undergo MV surgery due to high preoperative risk [12,17]. Recent data from prospective randomized clinical trials indicate that MV-TEER is now considered suitable for the general population, expanding its use beyond the previous focus on inoperable patients. The RESHAPE-HF2 trial demonstrated significant benefits of MV-TEER in treating severe mitral regurgitation compared to standard conservative treatment, particularly in terms of mortality and hospitalization rates [26]. Moreover, the MATTERHORN trial revealed that MV-TEER is on par with standard surgical treatment for severe MR when evaluated against a composite primary endpoint, which included mortality, heart failure-related hospitalizations, stroke, and secondary MV re-interventions or the implantation of assist devices [27]. In a meta-analysis of three controlled prospective randomized trials, Anker et al. demonstrated that MV-TEER significantly improves survival rates and reduces heart failure-related hospitalizations [28].

However, the pathophysiology of severe MR is complex and multifaceted. Patients with severe MR are accompanied by an increased preload of the LV impairing systolic function and thus CO, while the left atrium (LA) dilates and LA pressure increases [29]. Thus, severe MR also leads to increased pulmonary artery pressure and subsequent backward failure, resulting in elevated pulmonary vein congestion that burdens right ventricular function. This, in turn, exacerbates venous congestion, further impairing organ and tissue perfusion [30]. In the presence of coexisting CKD, the additional volume overload from renal insufficiency worsens venous congestion, which subsequently elevates cardiac preload, further straining cardiac systolic function and decreasing CO [30,31,32,33,34].

Regarding the results of the overall cohort in this study, the significant decrease in SVR and the improvement in renal function, indicated by an increase in eGFR, suggests reduced cardiac afterload and therefore enhanced tissue and organ perfusion—particularly renal organ perfusion—following MV-TEER in the 3–5-day follow-up. In correspondence, CO and CI also increased, depicting a noteworthy improvement in the hemodynamic situation after MV-TEER.

After dividing the patients into two subgroups based on their renal function (eGFR < 35 mL/min vs. eGFR ≥ 35 mL/min), a noteworthy improvement in CO and CI was particularly observed in the eGFR < 35 mL/min subgroup, whereas SVR dropped significantly and the glomerular filtration rate (eGFR) improved after MV-TEER. These findings indicate a significant enhancement of cardiac systolic function, leading to improved tissue perfusion and renal function, particularly in this subgroup. In the eGFR ≥ 35 mL/min subgroup, cardiac output (CO), cardiac index (CI), and eGFR also increased, while systemic vascular resistance (SVR) decreased; however, these changes did not reach statistical significance.

Based on our pathophysiological understanding, the improvement in cardiac function and tissue perfusion may be attributed to the sufficient reduction in regurgitant volume after MV-TEER, which may lead to a subsequent decrease in right ventricular (RV) and LV preload, ultimately benefiting global cardiac systolic function and alleviating venous congestion. Additional information on central venous pressure (CVP), pulmonary arterial pressure (PAP), and post-capillary wedge pressure (PCWP) could have further validated these results. Unfortunately, however, no invasive data were collected as part of this study.

A significant reduction in mitral regurgitation (MR) was achieved in our overall cohort, as well as in the eGFR < 35 mL/min and eGFR ≥ 35 mL/min subgroups, consistent with findings from previous studies and supporting the overall efficacy of MV-TEER in reducing MR [38,40,41]. Patients with eGFR < 35 mL/min, who were more decompensated before the procedure, showed remarkable early improvements in cardiac function and vascular resistance, suggesting that they may experience significant long-term benefits, although further studies are needed to confirm this.

Moreover, consistent with these results observed in our overall cohort as well as the eGFR subgroups, the impact of MV-TEER on quality of life demonstrated significant improvement as measured by the EQ-5D-3L questionnaire.

Overall, the data from this study highlight the benefits of MV-TEER in terms of hemodynamics, organ and tissue perfusion, and quality of life while achieving a substantial reduction in MR in all patients. The most significant improvements in hemodynamics and organ perfusion were observed in the subgroup of patients with an eGFR < 35 mL/min, highlighting the considerable potential for enhancement in this particularly vulnerable group. Consequently, it suggests that MV-TEER could be especially beneficial for patients with severe MR and advanced renal insufficiency, thus facilitating recompensation, stabilizing cardiac output, and preserving critical organ function, ultimately contributing to better prognoses for these patients.

## 5. Limitations

The limitations of this study include the relatively small cohort size and single-center design, which may affect the generalizability of the results. In addition, due to the study design, neither invasive hemodynamic parameters nor data on long-term outcomes such as serious adverse kidney events were collected. However, to the best of our knowledge, this is the first study to investigate the impact of MV-TEER on expanded hemodynamics while also assessing its effects on quality of life, particularly in a population with multiple comorbidities and complex pathophysiological conditions. Further prospective, randomized studies with larger and more diverse cohorts are needed to confirm the underlying pathophysiological changes before and after MV-TEER to provide additional insights for further optimization of therapy, patient selection, peri-procedural management, and long-term outcomes.

## Figures and Tables

**Table 1:** Demographics and baseline characteristics of the overall cohort and in eGFR < 35 mL/min and eGFR ≥ 35 mL/min subgroups. (Abbreviations: BMI: body mass index, aHTN: arterial hypertension, DM: diabetes mellitus, CKD: chronic kidney disease, CRT: cardiac resynchronization therapy, ICD: implantable cardiac defibrillator, CHD: coronary heart disease, COPD: chronic obstructive pulmonary disease. TAPSE: tricuspid annular plane systolic excursion, TKS’ lateral: lateral tricuspid annular tissue doppler pulse wave velocity.

**Demographics, Characteristics, and Comorbidities**				
	**Total** **(n = 45)**	**eGFR < 35 mL** **/min** **(n = 16)**	**eGFR ≥ 35 mL/min** **(n = 29)**	***p* Value**
Age (years)	78.7 ± 6.7	78.3 ± 7	78.9 ± 6.1	0.776
Female n (%)	18 (40)	7 (43.8)	18 (72)	0.237
Male n (%)	27 (60)	9 (56.3)	11 (37.9)	0.237
**BMI**	**26.5 ± 5**	**26.1 ± 4.4**	**26.8 ± 5.3**	**0.041**
History of aHTN n (%)	32 (71.1)	11 (68.8)	21 (72.4)	0.654
History of dyslipidemia n (%)	22 (48.9)	9 (56.3)	13 (44.8)	0.463
History of DM n (%)	15 (33.3)	4 (25)	11 (37.9)	0.378
Known history of CKD n (%)	29 (64.4)	13 (81.3)	16 (55.2)	0.080
Atrial fibrillation n (%)	35 (77.8)	11 (68.8)	24 (82.8)	0.279
CRT n (%)	10 (22.2)	4 (25)	6 (20.7)	0.739
**ICD n (%)**	**5 (11.1)**	**4 (25)**	**1 (3.4)**	**0.028**
CHD n (%)	33 (73.3)	12 (75)	21 (72.4)	0.851
COPD n (%)	12 (26.7)	7 (43.8)	5 (17.2)	0.054
LVEF (%) median [IQR] mean (SD)	55 [39–56] 47.5 ± 11.2	52 [35–56] 47.5 ± 11.5	55 [39–56] 47.4 ± 11.1	0.6
MR grade 3 n (%) 4 n (%)	25 (55.6) 20 (44.4)	9 (56.3) 7 (43.8)	16 (55.2) 13 (44.8)	0.095
MR regurgitation volume (mL)	59.5 [40–72.5]	53.5 [40.25–77.5]	59 [38–71.5]	0.924
MR EROA (cm^2^)	0.36 [0.23–0.59]	0,40 [0.22–0.68]	0.33 [0.24–0.54]	0.553
TAPSE (cm)	21 [19–22.5]	22 [20–23]	21 [18.5–22]	0.443
TKS’ lateral (cm/s)	12 [10.2–13]	12 [11.14.5]	11 [10–12.3]	0.184
**Hemoglobin (g/L)**	**115.4 ± 191**	**111.9 ± 18.5**	**117.7 ± 18.1**	**0.007**
Hematocrit (L/L)	0.36 [0.32–0.38]	0.35 [0.32–0.38]	0.36 [0.32–0.39]	0.537
Sodium (mmol/L)	139.3 ± 3.2	139.4 ± 3.03	139 ± 3.3	0.543
Potassium (mmol/L)	4.05 [3.65–4.35]	4.3 [3.8–4.9]	4.0 [3.6–4.1]	0.063
**NT-proBNP (pg/mL)**	3782 [1725–8513]	8513 [3859–15,356]	2046 [1378–4805]	<0.001
**Medication**				
Torasemide (mg/d) n = 45	15 [10–15]	15 [10–15]	15 [10–15]	1.0
Eplerenone (mg/d) n = 38	25 [25–25]	25 [25–25]	25 [25–25]	1.0
Bisoprolol (mg/d) n = 38	5 [2.5–5]	5 [2.5–5]	5 [2.5–5]	0.81
Ramipril (mg/d) n = 22	5 [5–10]	5 [5–10]	5 [5–10]	0.868
Candesartan (mg/d) n = 23	16 [16–24]	16 [16–22]	16 [16–32]	0.506
Dapagliflozine (mg/d) n = 36	10 [10–10]	10 [10–10]	10 [10–10]	1.0
Amlodipine (mg/L) n = 37	10 [5–10]	10 [5–10]	10 [5–10]	0.15
**Hemodynamics**				
CO (L/min)	4.38 ± 1.58	3.94 ± 1.6	4.63 ± 1.5	0.165
CI (L/min/m^2^)	2.36 ± 2.36	2.52 ± 0.82	2.07 ± 0.69	0.055
SVR (N × s/m^5^)	1596 [1177–2132]	1791 [1285–2612]	1514 [1138–2022]	0.129

**Table 2:** Changes in hemodynamics and renal function in the overall cohort after MV-TEER (Abbreviations: SAP: systolic arterial pressure, DAP: diastolic arterial pressure, MAP: mean arterial pressure, HR: heart rate, CO: cardiac output, CI: cardiac index, SVR: systemic vascular resistance, eGFR: estimated glomerular filtration rate, MR: mitral regurgitation).

	T1 (Pre-Procedure) (n = 45)	T2 (3–5 d Post-Procedure) (n = 45)	*p* Value
SAP (mmHg)	118.89 ± 18.86	114.02 ± 18.79	0.135
DAP (mmHg)	67.2 ± 11.65	66.09 ± 11.54	0.601
MAP (mmHg)	84.07 ± 12.59	81.84 ± 10.81	0.284
HR (bpm)	77.1 ± 26.8	80.1 ± 24.1	0.549
CO (L/min)	4.13 [3.09–5.38]	4.3 [3.86–5.69]	0.025
CI (L/min/m^2^)	2.31 [1.82–2.78]	2.44 [2.11–2.82]	0.032
**SVR (N × s/m^5^)**	**1765 [1177–2132]**	**1427 [2226–3876]**	**0.003**
**eGFR (mL/min)**	**46.26 ± 21.56**	**50.38 ± 21.34**	**0.03**
**MR Grade**	**3 [3–4]**	**1 [1–1]**	**<0.001**

**Table 3:** Changes in hemodynamics and renal function in the eGFR < 35 mL/min and eGFR ≥ 35 mL/min subgroups (Abbreviations: SAP: systolic arterial pressure, DAP: diastolic arterial pressure, MAP: mean arterial pressure, HR: heart rate, CO: cardiac output, CI: cardiac index, SVR: systemic vascular resistance, eGFR: estimated glomerular filtration rate, MR: mitral regurgitation).

	eGFR < 35 (T1) n = 16	eGFR < 35 (T2) n = 16	*p* Value	eGFR ≥ 35 (T1) n = 29	eGFR ≥ 35 (T2) n = 29	*p* Value
SAP (mmHg)	119.56 ± 14.36	117.13 ± 16.5	0.599	118.52 ± 21.18	112.21 ± 20.01	0.161
DAP (mmHg)	68.89 ± 7.53	67.44 ± 9.85	0.579	66.28 ± 13.43	65.34 ± 12.47	0.758
MAP (mmHg)	85.5 ± 6.82	84 ± 8.85	0.551	83.28 ± 14.92	80.66 ± 11.73	0.375
HR (bpm)	74.6 ± 18	74.7 ± 13.3	0.972	78.48 ± 30.77	83.07 ± 28.19	0.546
CO (L/min)	3.94 ± 1.6	4.47 ± 1.72	0.035	4.38 [3.27–5.52]	4.63 [3.93–5.86]	0.247
CI (L/min/m^2^)	2.07 ± 0.69	2.34 ± 0.68	0.031	2.43 [2.01–2.89]	2.53 [2.2–3.039]	0.294
SVR (N × s/m^5^)	1791 [1285–2612]	1618 [2205–5654]	0.007	1514 [1138–2022]	1397 [2233–3793]	0.061
eGFR (mL/min)	25.63 ± 6.54	33.81 ± 16.05	0.018	57.66 ± 18.15	59.51 ± 18.38	0.408
MR Grade	3 [3–4]	1 [1–1]	<0.001	3 [3–4]	1 [1–1]	<0.001

**Table 4:** Impact of MV-TEER on quality of life based on the EQ-5D-3L data. Comparison of parameters 24 h before MV-TEER (T1) and 3 months after the procedure (3M-FU). In the overall cohort as well as in the eGFR < 35 mL/min and eGFR ≥ 35 mL/min subgroups, a substantial improvement in quality of life could be observed (VAS: visual analogue scale).

	Overall Cohort		
	T1	3M-FU	*p* Value
EQ-VAS (%)	51.7 ± 0.18	62.9 ± 0.17	*p* < 0.001
EQ-5D-3L index value	0.44 ± 0.39	0.66 ± 0.20	*p* < 0.001
	**eGFR < 35 mL/min**		
EQ-VAS (%)	51.6 ± 0.14	60.6 ± 0.18	0.015
EQ-5D-3L index value	0.527 ± 0.13	0.61 ± 0.19	0.034
	**eGFR ≥ 35 mL/min**		
EQ-VAS (%)	51.8 ± 0.17	64 ± 0.17	<0.001
EQ-5D-3L index value	0.475 ± 0.4	0.68 ± 0.19	<0.001

## Data Availability

Data will be made available upon individual request. Since the data is sensitive and may require additional protection or contextual restrictions, we have decided not to make it publicly available in a database. Instead, we offer access on an individual request to ensure that it is used and interpreted appropriately.

## Supplementary Materials

The following supporting information can be downloaded at: https://www.mdpi.com/article/10.3390/biomedicines12112648/s1, Table S1: Univariate linear regression analysis of MAP, SAP and DAP as predictor variables with SVR at T1. (Abbreviations: MAP: mean arterial pressure, SAP: systolic arterial pressure, DAP: diastolic arterial pressure, 95% CI: 95% confidence interval); Table S2: Univariate linear regression analysis of MAP, SAP and DAP as predictor variables with SVR at T2. (Abbreviations: MAP: mean arterial pressure, SAP: systolic arterial pressure, DAP: diastolic arterial pressure, 95% CI: 95% confidence interval)
