# Supplementary material for: Mitral Valve Transcatheter Edge-to-Edge Repair (MV-TEER) in Patients with Secondary Mitral Regurgitation Improves Hemodynamics, Enhances Renal Function, and Optimizes Quality of Life in Patients with Advanced Renal Insufficiency
Source: Biomedicines. 2024 Nov 20;12(11):2648. doi: 10.3390/biomedicines12112648 (PMC11591953; doi:10.3390/biomedicines12112648)
Supplement: Supplementary file 1 [file biomedicines-12-02648-s001.zip › biomedicines-3291874-supplementary.pdf]

Table S1: Univariate linear regression analysis of MAP, SAP and DAP as predictor variables with SVR at T1. (Abbreviations: MAP: mean arterial pressure, SAP: systolic arterial pressure, DAP: diastolic arterial pressure, 95% CI: 95% confidence interval)

	p value	Regression coefficient B	95% CI
MAP T1	0.036	19.17	1.321-37.015
SAP T1	0.091	10.41	-1.73-22.54
DAP T1	0.052	19.29	-0.15-38.73

Table S2: Univariate linear regression analysis of MAP, SAP and DAP as predictor variables with SVR at T2. (Abbreviations: MAP: mean arterial pressure, SAP: systolic arterial pressure, DAP: diastolic arterial pressure, 95% CI: 95% confidence interval)

	p value	Regression coefficient B	95% CI
MAP T2	0.024	33.1	4.48-61.7
SAP T2	0.218	10.65	-6.65-27.81
DAP T2	0.037	28.82	1.77-55.9
